# Comparison of Supercritical CO_2_-Drying, Freeze-Drying and Frying on Sensory Properties of Beetroot

**DOI:** 10.3390/foods9091201

**Published:** 2020-08-31

**Authors:** Nikola Tomic, Ilija Djekic, Gerard Hofland, Nada Smigic, Bozidar Udovicki, Andreja Rajkovic

**Affiliations:** 1 Department of Food Safety and Quality Management, Faculty of Agriculture, University of Belgrade, Nemanjina 6, 11080 Belgrade, Serbia; idjekic@agrif.bg.ac.rs (I.D.); nadasmigic@agrif.bg.ac.rs (N.S.); bozidar.udovicki@agrif.bg.ac.rs (B.U.); arajkovic@agrif.bg.ac.rs (A.R.); 2 FeyeCon Carbon Dioxide Technologies, Rijnkade 17A, 1382 GS Weesp, The Netherlands; gerard.hofland@feyecon.com; 3 Department of Food Technology, Food Safety and Health, Faculty of Bioscience Engineering, Ghent University, Coupure Links 653, B-9000 Ghent, Belgium

**Keywords:** supercritical CO_2_-drying, beetroot snacks, preference mapping, mean drop analysis

## Abstract

The aim of this study was to compare the sensory quality and acceptance of dried ready-to-eat beetroot snacks as a result of different drying methods applied: supercritical CO_2_-drying (scCO_2_-drying), frying, and freeze-drying. Descriptive sensory analysis, quality rating (10 assessors), and consumer acceptance testing (*n* = 102) were performed. Mean overall quality scores within the range of “very good” quality were found only in non-precooked scCO_2_-dried samples which were characterized by typical magenta color, low level of shape and surface deformations, pronounced brittleness and crispiness, and good rehydration during mastication. The other samples were in the range of “good” quality. The pre-cooking step before scCO_2_-drying negatively influenced the sensory quality parameters, particularly appearance. Around 60% of tested consumers showed a preference for the fried and non-precooked scCO_2_-dried samples. The drivers of liking were mostly related to the characteristics of the product, which was salted, fried, and crispy, with an oily and overburnt flavor, i.e., the product most similar to commercial potato chips products. Freeze-drying had a negative effect primarily on appearance and flavor. According to the sensory evaluation conducted, direct scCO_2_-drying without a pre-cooking step showed itself as a promising alternative drying technology in the production of dried beetroot snacks.

## 1. Introduction

Drying of biological material is a controlled effort to preserve the structure or create a new one that serves for functional purposes [1]. The main technological objectives of food drying are [2]: preservation; reduction in weight and volume; transport and storage facilitation; and achieving a desirable sensory profile of different flavors, chewiness, crispiness, firmness, etc.

The food product’s microstructure is often negatively affected by the movement and ultimate loss of water during the drying process which influences physical properties, nutritional availability, and also chemical and microbiological stability [3,4]. Consumers nowadays demand high-quality, nutritious, fresh, convenient, additive-free, safe food products with a natural flavor and taste, and an extended shelf-life [5,6,7]. To achieve dehydrated fruit or vegetable commodities of high quality at a reasonable cost, in the sense of minimizing the loss of volatiles, loss of flavors, changes in texture, changes in color, and also a decrease in nutritional value [5], dehydration must occur fairly rapidly [8]. Enhancing drying rates has been a major challenge for food engineers [1]. Air-drying, which is still the most commonly used drying operation in the food industry [3,5,9], can bring many reconstituted products, such as powdered milk or dry pasta, with sensory profiles very similar to the original material. Further, certain air-dried commodities such as grains and legumes show desirable textural characteristics after cooking due to high rehydration capability, but the structure of most fruits and vegetables is usually negatively affected during air-drying resulting in poor reconstitution properties when compared to their fresh state [1]. Structural changes are important since food texture, as a complex sensory attribute which can be perceived and described only by human beings, is directly influenced by the structure of food, i.e., it derives from the structure (molecular, microscopic, or macroscopic) of food [10]. A decrease in the sensory quality [5] influenced by application of higher temperatures during air-drying (typically 65–85 °C) is most often reflected in the small volume, great shrinkage, high density, low porosity, and increased hardness of the dried product [4,11]. Heat can also affect the color of the dried commodity. As a part of the cellular structure, betalains are the main red pigments in beetroot. These compounds are very sensitive to heat, light, and oxygen, and therefore air-drying triggers the oxidation of betalains in beetroot due to prolonged exposure to higher temperatures [12].

Although primarily considered as a thermal preservation technique, according to Oreopoulou et al. [13], frying can be defined as a process of cooking and drying through contact with hot oil. Its preservative effect is reflected through both thermal destruction of microorganisms and enzymes and a reduction in water activity at the surface or throughout the product. Food products that are fully dried during the frying process, such as potato chips or other extruded snacks, have a shelf-life up to several months, which is usually limited by quality deterioration of the absorbed oil and development of a rancid odor and flavor. During food frying, heat is transferred by convection from the surrounding oil to the surface of the food and by conduction within the solid food. Mass transfer is reflected through the vaporization of water from the surface of the product and on the other side through the penetration of oil into the food. Along with water removal, the surface of the product becomes more and more dry causing the formation of a crust. Considering the process temperature and time, in general, the lower the temperature and the longer the process time, the slower the cooking rate of the product, the firmer the crust and texture, and the higher the oil content absorbed by the product [13]. The unique texture–flavor combination of fried snack foods makes them highly appreciated within different categories of consumers.

Freeze-drying is considered as a superior drying method for many fruits and vegetables and the best solution for food drying in general, which can deliver dried products of high commercial value with good sensory quality and a high level of nutrients retention [8,14]. Since the process implies a direct transition from solid to gaseous state without melting and without exposing the product to high temperatures, the structure of the product remains porous which facilitates rapid product rehydration, structural changes and shrinkage are largely avoided, movement of the soluble solids is minimized, thermal damage is minimized, and flavor, color, and appearance are highly preserved [8,15]. On the other side, freeze-drying is a highly energy-consuming technology with high capital costs which limits its industrial application to high added value products such as baby foods, instant products (coffee, soups), exotic fruits and vegetables, or mushrooms [16].

Application of supercritical fluids (SCFs) as extraction solvents in the food industry dates back to the mid-20th century [9,17]. Being at a temperature and pressure above the critical point, SCFs have the special combination of liquid-like solvating properties and density, and gas-like diffusivity and viscosity which makes them excellent solvents [18], while the fluid can be recovered and reused. Carbon dioxide is the most intensively used agent in supercritical processes applied to food [19]. CO_2_ is nontoxic, non-carcinogenic, non-flammable, odorless, colorless, and thermodynamically stable, with the possibility of adjusting its thermophysical properties, such as viscosity, diffusivity, density, or dielectric constant, by varying the temperature and/or pressure [18], and its usage in food industry is approved without declaration [19]. The removal of water during supercritical CO_2_-drying is not based on sublimation or vaporization, but on dissolving water in the scCO_2_ [9]. Due to these gas–liquid properties of scCO_2_, the structure of the dried material remains preserved to a high extent. Problems that occur with conventional air-drying processes, in which the solid structure can collapse due to surface tension effects at the vapor–liquid interfaces, are absent in the case of scCO_2_-drying [3,17]. Further on, due to the low critical parameters of CO_2_ (304.1 K, 7.38 MPa) [20], the drying process can be conducted at relatively low temperatures (e.g., 40 °C). The main drawback is the relatively low solubility of water in scCO_2_ (2.5 mg/g at 40 °C, 20 MPa) [3,21]. The drying conditions are most often reflected in the application of scCO_2_ under pressure within the range of 10 to 14 MPa and temperature between 40 and 60 °C with the fluid flow rate of 80–220 kg/h [22,23,24]. The drying time that must be ensured, in order to achieve an appropriate level of dehydration of a plant material under these conditions, ranges from 6 to 16 h [24]. According to the findings of Zambon et al. [24], the most influential process variable in reaching an appropriate level of water activity is temperature, as it acts directly on the water solubility in scCO_2_. The pressure significantly influences the drying efficiency only at lower temperatures and a longer drying time [24]. For the purpose of commercial drying, scCO_2_ is widely used for the drying of gels [25] and also there are studies that reported its practical application for the preservation of decellularized esophageal scaffolds [26]. Application of scCO_2_ in food drying is still limited to few products at the small pilot scale [22,24,27,28,29] and additional studies are still needed for scCO_2_-drying technology to be developed at industrial scale. The pilot scale scCO_2_-drying unit used in this study still has a low technology readiness level and requires further work in order to reach industrial application [30], first of all to validate its effectiveness in various working regimes [31].

Previous studies reported that the application of scCO_2_ showed a potent antimicrobial effect against both bacteria and fungi [32,33,34] which allows scCO_2_-drying to be used as a promising “green” technology than can combine drying and pasteurization in one single step. On the other side, recent studies showed that scCO_2_-drying can bring and retain, for at least six months, dried apple slices with similar sensory quality and consumer acceptance levels as obtained by freeze-drying, in the case of using non-permeable materials and an inert atmosphere for packing the products [22,23].

The aim of this study was to investigate the effects of the supercritical CO_2_ drying method, as compared to the freeze-drying and frying techniques, on the sensory quality and acceptance of dried beetroot cuts intended to be eaten as ready-to-eat snacks.

## 2. Materials and Methods

### 2.1. Dried Beetroot Samples

**Figure 1:**
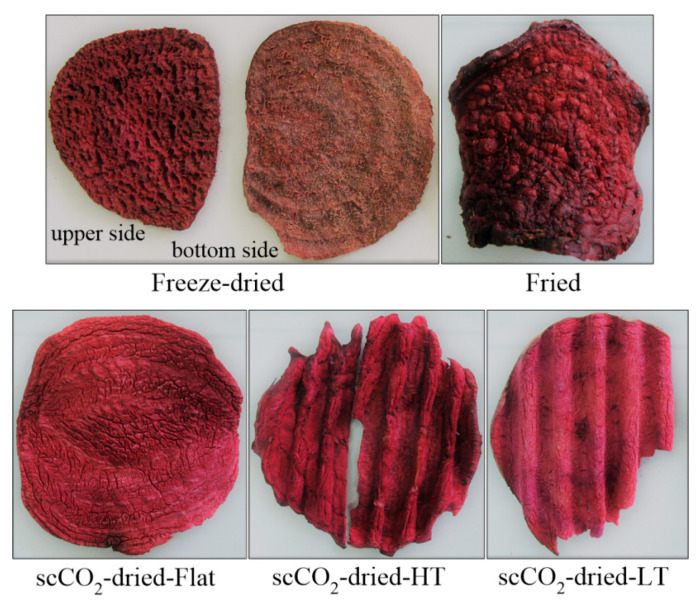
Images of the dried beetroot samples used in this study. Supercritical CO_2_-dried-HT sample was cooked in boiling water before drying, the rest were subjected to the drying processes in a raw state. The upper and bottom sides of a beetroot chip substantially differed from each other in appearance only in the case of freeze-dried samples.

Fresh red beetroot (*Beta vulgaris*) was purchased at a local market in the Netherlands (Kruythof aardappelhandel, 3291 CN Strijen, The Netherlands). After thorough washing, beetroot was sliced without removing the skin into flat or “wavy” circular cuts of relatively similar size (around 3 mm in thickness). Three different drying methods were applied: supercritical drying using CO_2_ (scCO_2_-drying), freeze-drying, and frying. ScCO_2_-drying was performed in a patented equipment [35] under a pressure of 10.0 MPa at 40 °C for 14 h. ScCO_2_-drying was combined with a pre-cooking step, and also two types of cutting were applied to obtain flat and wavy beetroot discs, in which way three experimental scCO_2_-dried samples were obtained: scCO_2_-dried-Flat (not pre-cooked; raw sliced, flat cuts); scCO_2_-dried-LT (not pre-cooked; raw sliced, wavy cuts); and scCO_2_-dried-HT (pre-cooked in boiling water and sliced; wavy cuts). “Wavy” shape was introduced in order to examine the influence of this type of appearance on consumer acceptance. Freeze-drying of flat raw beetroot cuts was performed in a 20-L freeze-dryer SuperModulyo (Thermo Scientific, Waltham, MA, USA) within 48 h. The pressure during sublimation was kept at 20 Pa and during desorption at 5 Pa, while the temperature of −25 °C during sublimation was gradually increased up to 40 °C during desorption. Deep fat frying of flat beetroot cuts was done in boiling vegetable oil at 160 °C for 3.5 min (raw beetroot cuts were first salted with around 25 g of salt per 1 kg of beetroot and then fried). Water activity of the dried samples was 0.32 ± 0.02. Appearance of the beetroot samples used in the study is shown in Figure 1.

After drying, the samples (100–200 g) were packed under 100% N_2_ in aluminum-polyethylene (Alu-PE) pouches (thickness: 98 μm, aluminum layer: 8 μm; Goglio S.p.A, Milan, Italy) and placed in the dark at ambient temperature (≈22 °C). All of the beetroot samples were produced within two separate batches (two replications). The samples were evaluated between the third and fourth week upon packing.

### 2.2. Sensory Analysis

The sensory tests were conducted in the sensory testing laboratory at the University of Belgrade. Testing conditions such as sample serving procedures, instructions to panelists, palate cleansing, and training sessions are described in Tomic et al. (2019) [22]. In short, random 3-digit numbers were used for the sample-coding, the panelists used low-sodium mineral water for palate cleansing, no strict instructions were given to the panelists regarding the swallowing/expectorating of individual bites, additional beetroot samples were kept in closed glass jars for the purpose of orthonasal olfaction during evaluation. Environmental conditions during testing were in accordance with ISO 8589:2007 (Sensory analysis—General guidance for the design of test rooms) and ISO 11136:2014 (Sensory analysis—Methodology—General guidance for conducting hedonic tests with consumers in a controlled area). The trained panelists who participated in descriptive analysis and quality judging were of good general health, of normal range for BMI of 18–25 kg/m^2^, with no dental wearers and reported dental problems, which is in line with the recommendations of Forde et al. [36]. For this particular reason, the descriptive/objective quality sensory panel had six 2-h additional training sessions in total prior to the analyses. These calibration trainings were performed using different types of dried commodities (bell pepper, apple, and beetroot). Considering consumer sensory testing, all volunteers gave verbal consent that they were of good general health with no dental issues before conducting the test.

#### 2.2.1. Descriptive Sensory Testing

Descriptive sensory testing was performed by a 10-university members sensory panel (4 men and 6 women) experienced in fruits and vegetables quality judging.

The testing was done in two replications by assessing the intensity of 20 sensory attributes using 15 cm line-scales with verbal anchors at both ends. Since there were no reference standards, the assessors were using the scales in their own way by comparing the samples to each other [37,38]. The list of the attributes with terminal verbal anchors is shown in Table 1. A Latin square design was used in the samples’ presentation scheme.

#### 2.2.2. Sensory Quality Rating

Sensory quality rating was also done in two replications by the same assessors used in the descriptive analysis.

The evaluation was conducted using a 5-level quality scoring system described in Djekic et al. (2018) [23] supported by the internal laboratory guidelines for fruits and vegetables quality judging. By dividing each of the five integer quality scores into quarters, the 0–5 score range was transformed into a 20-responses category scale. Four groups of sensory attributes were evaluated in order to assess overall sensory quality: appearance, flavor, texture, and odor. In order to distinguish the selected sensory attributes according to their impact on overall quality, each attribute was assigned an appropriate correction factor (CI—coefficient of importance): 2, 8, 6, and 2, respectively. Overall sensory quality score was calculated by multiplying individual scores with appropriate CIs and by dividing the sum of the obtained corrected scores by the sum of CIs.

#### 2.2.3. Consumer Sensory Testing

Consumer acceptance tests were performed by 103 students from the university (102 responses in total were further processed: 43 males and 59 females). Students between the ages of 19 and 25 were randomly selected and were chosen if they were relatively frequent consumers (more than two times per month) of dried fruits/vegetables (regardless of direct consumption of the products as snacks or through other food products and meals such as cereal breakfasts/bars).

A 9-point hedonic scale [39,40] was used to evaluate overall acceptance, appearance acceptance, as well as flavor acceptance. Further on, just-about-right (JAR) scales of 9 points (1 = too little, 5 = JAR, 9 = too much) [41] were used to assess the samples for accepted intensities of “hardness”, “chewiness”, “crispiness”, “color”, “saltiness”, and “bitterness” (only “too much” part of the scale).

### 2.3. Statistical Analysis

#### 2.3.1. Descriptive Data and PREFMAP

The significance of the multivariate effect for samples was tested by multivariate analysis of variance (MANOVA) with “samples” as the fixed factor. After that, three-way ANOVA (followed by Tukey’s HSD test) was conducted in order to identify those characteristics that significantly discriminate among the tested products (“samples” = fixed factor; “assessors” and “replications” = random factors). Original descriptive data were first standardized for each assessor before applying both MANOVA and ANOVA. As a result, two sensory characteristics (“musty odor” and “denseness”) were excluded from subsequent dimensional reduction analysis (Table 1) since they did not significantly discriminate (*p* < 0.05) among the samples. Original descriptive data for the attributes that remained after removing the two, divided into personal data matrices, were subjected to generalized Procrustes analysis (GPA). Then, principal component analysis (PCA) was applied to the obtained consensus data. External preference mapping (PREFMAP) [42] was done by applying linear multiple regression analysis in which the extracted PC space was regressed against the overall hedonic data. K-means cluster analysis was used to segment the obtained regression coefficients.

#### 2.3.2. Quality Data

Three-way ANOVA was applied on raw quality data (“samples” = fixed factor; “assessors” and “replications” = random factors) together with Tukey’s HSD post hoc test.

#### 2.3.3. Consumer Data

In order to examine the differences both between the consumer clusters obtained by K-means cluster analysis in PREFMAP (within the samples) and between the samples within the clusters, raw hedonic data were subjected to ANOVA (“samples” = fixed factor).

One-way ANOVA was also performed in order to test for gender differences in hedonic scores.

Mean drop analysis was done as described by Schraidt (2009) [43]. In brief, for each sensory attribute evaluated by using the appropriate JAR scale, the tested consumers were first grouped into three categories according to their JAR scores: 1, 2, and 3 = “below JAR (i.e., too little of an attribute)”; 4, 5, and 6 = “at JAR”; and 7, 8, and 9 = “above JAR (i.e., too much of an attribute)”. The mean overall hedonic scores were calculated for each category and then the mean drops were obtained by subtracting the mean hedonic score of each non-JAR category from the mean of the JAR category. Statistical significance of the mean drops was tested by applying ANOVA and Tukey’s HSD test. The cutoff was set at 20% of the total number of respondents.

#### 2.3.4. Software

The statistical analyses were performed using both Idiogrid software version 2.4/2008 (Oklahoma State University, Stillwater, OK, USA) [44] (GPA, PCA) and SPSS Statistics 17.0 (IBM, Armonk, NY, USA) (MANOVA, ANOVA, PCA), at a 0.05 level of statistical significance.

## 3. Results and Discussion

### 3.1. PREFMAP

Generalized Procrustes analysis of the descriptive data showed strong agreement among the assessors and replications (consensus proportion = 0.94; *p* < 0.05) with relatively small differences in overall variability of the individual data matrices (isotropic scaling values ranged from 0.72 to 1.44 which are relatively close to 1) [45]. Upon PCA of the consensus data matrix, 18 original variables fit into the new four-dimensional PC space. The Kaiser criterion [46] was used for making a decision on the number of PCs that should be retained for the overall variability explanation (6.9, 4.0, 4.0, and 3.1, PC-1 to PC-4, respectively).

**Figure 2:**
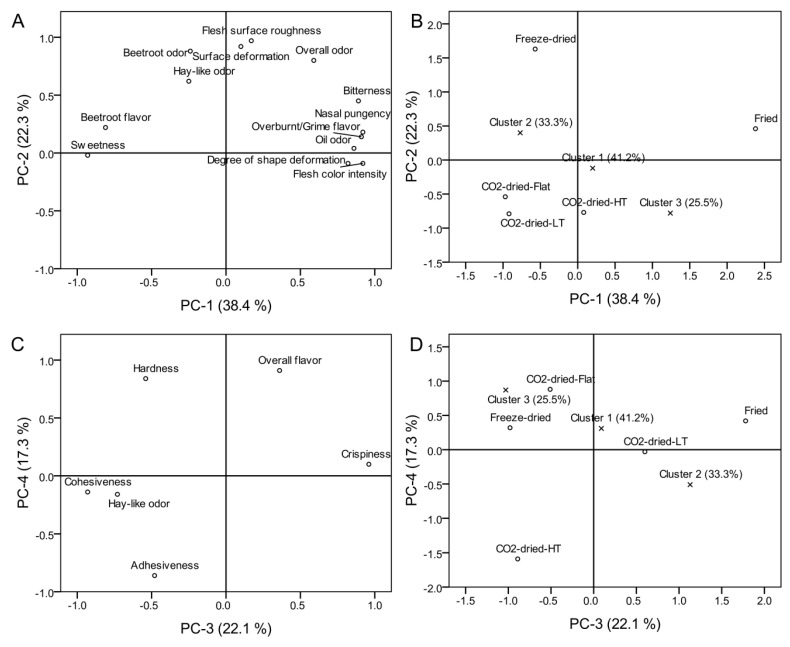
Four-dimensional principal component-space (PC-1 to PC-4) of the sensory descriptive data (10 assessors × 2 replications) of dried beetroot samples, previously subjected to generalized Procrustes analysis, regressed against the overall hedonic data (PREFMAP). (**A**,**C**): loading plots; (**B**,**D**): scores plots. Loadings cutoff was set at 0.60. Consumers (N = 102) are grouped within the three clusters. Samples abbreviations: “Flat” = not pre-cooked, flat cuts; “LT” = not pre-cooked, wavy cuts; and “HT” = pre-cooked, wavy cuts.

Figure 2 shows the PC space of the first four principal components extracted. The freeze-dried beetroot sample was characterized by the presence of various relatively large holes and cracks on the upper surface of the cuts (the side which was free during the drying), highly pronounced flesh surface roughness, pronounced beetroot and hay-like odor, pronounced cohesiveness, and lower level of crispiness. Surface deformation and flash surface roughness were the most pronounced in the freeze-dried sample when compared to the rest of the evaluated products (*p* < 0.05). The fried sample, on the far right side of both score plots (Figure 2), was characterized by a pronounced over-burnt/grime flavor, oil odor, nasal pungency, the highest degree of shape deformation (*p* < 0.05), bitterness, and lower levels of sweetness, beetroot flavor, and cohesiveness. This sample was also the darkest one in color, and with the highest levels of crispiness and bitterness (*p* < 0.05). The surface lumpiness observed in fried samples (Figure 1) originates from vapor bubbles entrapped under the formed crust on the very surface of the snack product during frying [13]. As it was expected, oil notes were noticed only in the fried sample. Certain characteristics that were typical for the fried sample, such as over-burnt flavor, nasal pungency, and shape deformation, were also noticed in the precooked scCO_2_-dried-HT sample. According to sweetness, three homogenous (*α* = 0.05) subsets of the samples without overlaps emerged (ANOVA data not shown) with increasing sweetness in the following order: (i) fried; (ii) scCO_2_-dried-HT; (iii) freeze-dried, scCO_2_-dried-Flat, and scCO_2_-dried-LT. It seems that the application of high temperatures during or before the drying process influences the perception of sweetness in dried beetroot. In addition, hay-like odor and common dried vegetables-like odor were at the highest level in the freeze-dried and scCO_2_-dried-HT samples as compared to the rest (*p* < 0.05), while totally absent in the fried beetroot cuts. The ScCO_2_-dried-HT sample differed to a certain extent in sensory profile from the other scCO_2_-dried samples which were not thermally treated before drying. Beside the mentioned flavor notes, the scCO_2_-dried HT sample was less hard, less cohesive, less crispy, and more adhesive (*p* < 0.05) than the other two which were characterized by a pronounced beetroot flavor, sweetness, less surface deformations and flesh surface roughness, and also shape deformations to a lesser degree.

Individual consumer overall hedonic scores (N = 102) were regressed against the four PCs. Cluster analysis revealed three consumer clusters with individual proportions of tested consumers greater than 20% (Cluster 1 = 41.2%; Cluster 2 = 33.3%; and Cluster 3 = 25.5%). The clusters are mapped within the PC space (Figure 2) by averaging the regression coefficients across the clusters. The results of the hedonic acceptance testing with the scores averaged across the clusters are shown in Table 2. Cluster 3 (25.5%) showed a preference for the scCO_2_-dried-Flat (7.5 ± 1.3) and fried (7.3 ± 2.1) beetroot samples, while Cluster 2 (33.3%) preferred the scCO_2_-dried-LT (7.5 ± 1.5) and fried (6.5 ± 2.7) samples. This means that around 59% of the tested consumers showed a preference for the scCO_2_-dried beetroot samples not subjected to the cooking step before the drying process. Similar results related to consumer acceptance were reported for scCO_2_-dried red bell pepper and apple fruits [22,24]. ScCO_2_-dried red bell pepper was assessed as acceptable by more than 60% of the tested consumers [24]. Preference of the consumer within Cluster 1 cannot be explained in this four-dimensional PC space since the position of the cluster is mainly close to the origin in the plots (PC1 to PC4 score values were less than 0.35). According to the average overall and flavor hedonic scores (6.6 and 6.2, respectively), the consumers within Cluster 1 slightly preferred also the fried sample (the rest of the scores were less than 6). It appeared that, in general, tested consumers showed their preference for the beetroot product which was salted, fried, crispy, with an oily and overburnt flavor, in other words, most similar to commercial potato chips products. Freeze-dried and scCO_2_-dried-HT samples did not gain the consumers’ attention and were scored with relatively low (<6) overall and flavor hedonic scores (Table 2). It seems also that the “wavy” shape of the scCO_2_-dried-LT and scCO_2_-dried-HT samples did not have an influence on the shape acceptance scores of the scCO_2_-dried beetroot products. Taking into account that descriptive analysis and quality judging showed that precooked scCO_2_-dried-HT beetroot wavy discs suffered shape deformations, the mean shape acceptance scores for the scCO_2_-dried-LT sample were not statistically higher within any consumer cluster when compared with the scCO_2_-dried-Flat beetroot sample.

Acceptance ratings of the total number of tested consumers, measured by using the hedonic scales, were also compared in order to assess the gender effect. The gender of the tested consumers did not influence liking for the examined dried beetroot snacks. Analysis of variance showed that there were no statistically significant differences (*p* > 0.05) between females and males within any of the examined modalities: overall, appearance, and flavor liking (data not shown).

Beside acceptable appearance and palatability, it is also expected of dried ready-to-eat agricultural commodities such as fruits and vegetables to preserve their nutritional content [47]. Since beetroot is rich in valuable active compounds such as carotenoids, betalains, polyphenols, and flavonoids, and also saponins, it has attracted significant scientific and consumer attention in recent years as a health-promoting functional food product [12]. As being mostly strong antioxidants, these compounds are sensitive to the promoters of oxidation such as oxygen, light, and heat. Due to prolonged exposure to elevated temperatures typical for hot air-drying (65–85 °C), in the presence of oxygen, oxidation and degradation of these compounds are inevitable, which causes the loss of their nutritional and health values [48,49]. Within the optimal pH range, temperature is the most influential factor for betalains degradation [50], betalains being the main red pigments in beetroot with intense antibacterial and antiviral activity that comes from their strong antioxidant potential [51]. Relatively low temperatures (e.g., 40 °C) observed in the case of using scCO_2_ for drying purposes are recognized as a nutritionally friendly preservation technology meeting these consumers’ demands [52]. In addition, reduced water activity, inactivation of enzymes, and increased light impermeability of dried plant tissue can decrease the sensitivity of these phytochemicals as compared to the raw state [12], as it was shown in the case of betalains in beetroot [50]. Stability of these kinds of products during storage can be increased by using protective packaging materials and an inert gas atmosphere for packing. According to the findings of Tomic et al. [22], scCO_2_-drying can bring and retain the same acceptance level of dried apples for at least 6–12 months as it can be obtained by the freeze-drying process provided the products were packed in the packaging material with low gas permeability (such as aluminum-polyethylene pouches used in their study) under an inert atmosphere (N_2_).

### 3.2. Mean Drop Analysis

**Figure 3:**
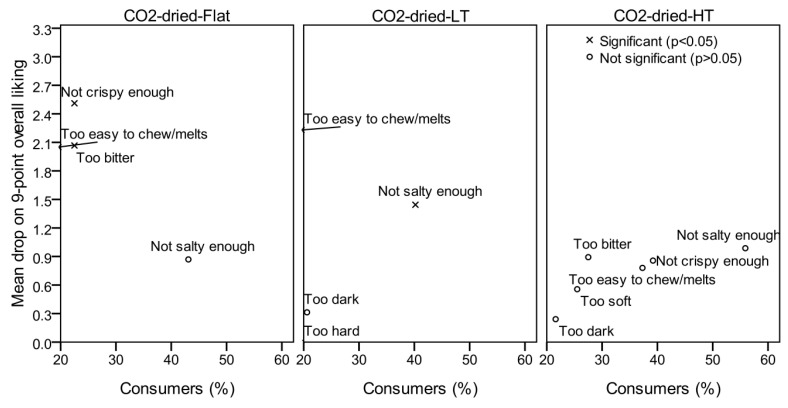
Mean drop analysis for supercritical CO_2_-dried beetroot samples (N = 102 respondents).

The results of the mean drop analysis are shown in Figure 3 only for the scCO_2_-dried beetroot samples.

Points in the mean drop plot represent the drops of the averaged hedonic scores linked to the consumer groups who felt a particular attribute “too little” or “too much” in regard to the “just about right” level. The direction of potential product modification is sought among those points with a large proportion of consumers and a statistically significant mean drop [39]. There were two large consumer groups (≥20%) with statistically significant (*p* < 0.05) mean drops (Figure 3), one of which felt the scCO_2_-dried-Flat sample was “not crispy enough” (22.5%), and one who felt the dried product was “too bitter” (22.5%). The scCO_2_-dried-LT sample was felt by the consumers as “not salty enough” (40.2% of the consumers, *p* < 0.05). As for the scCO_2_-dried-HT sample, there were no points in the plot with large consumer groups and significant mean drops, but it is worth mentioning that all of the mean acceptance scores for the “just right” consumer groups were less than 5.4 (data not shown), i.e., not in the range of acceptable products. The freeze-dried sample was rated as “too light/pale” in color, “not crispy enough” and “too bitter”, while the fried sample appeared to be felt as “too hard” and “too bitter” according to the tested consumers.

### 3.3. Sensory Quality Rating

Table 3 shows the results of the sensory quality rating of the dried products. Mean overall quality scores in the range of “very good” quality (i.e., above 3.5) were found only in the scCO_2_-dried-Flat and scCO_2_-dried-LT samples (3.7 both). Overall sensory quality levels of the other samples were in the range of “good” quality with the scores significantly lower as compared to the former two (*p* < 0.05). The non-pre-cooked scCO_2_-dried samples (Flat and LT) were characterized by the typical beetroot magenta color evenly distributed over the surface to a large extent, low level of shape and surface deformations, non-intensive beetroot odor and flavor, pronounced brittleness and crispiness, and good rehydration during mastication. The pre-cooking step before drying negatively influenced the sensory quality parameters, at first appearance. The scCO_2_-dried-HT sample appeared shrunken and distorted, the waves obtained after initial slicing with the curved knife were mostly damaged after drying, the surface was darker in color with the occurrence of dark discolorations as compared with the other two scCO_2_-dried products, flavor was empty and hay-like with the beetroot flavor totally lost, and it was harder, more cohesive, and less airy than the Flat and LT scCO_2_-dried samples. Similar negative effects of scCO_2_-drying on sensory quality were observed also in red bell pepper and apple fruits. In the case of red bell pepper, the main defects were related to the loss of color and flavor intensities, with the simultaneous occurrence of a hay-like odor [24], while the apples suffered partial shape deformation, the appearance of cracks on the flesh surface, and the appearance of reddish/pinkish discoloration in flesh originating from the skin color [22,23]. Satisfied textural characteristics such as crispiness, airy denseness, good chewiness, and good rehydration during mastication were observed in both pepper and apple cases. Frying of beetroot slices led to the product being characterized by pronounced shape deformation (mostly bended in the shape of a saddle), very dark in magenta color with the grime-black areas on the surface, the presence of airborne blisters on the surface indicating that the product was oil-fried, pronounced oil-like odor and over-burnt/grime flavor, bitterness, and mild sourness. On the other hand, the fried product was very crispy, firm, but not too hard or brittle, with a relatively good rehydration rate during chewing (with residual small solid particles in the mass), and accordingly the texture was assessed as “very good” (4.1 ± 0.8, Table 3). Contrary to expectations based on the experience with red bell pepper and apple in the previous studies [23,24], freeze-dried beetroot got relatively low quality scores in general. The score-lowering factors in the case of the freeze-dried sample were primarily related to the appearance attributes such as the presence of numbers of relatively large holes and cracks on the upper surface of the beetroot slices, uneven distribution of magenta color on the upper surface with the presence of discolorations and ash-gray color notes which are not characteristic of beetroot, absence of magenta color on the bottom surface of the cuts with dominating dark brownish-ash-gray color, but also to less intensive beetroot flavor with the presence of the common root vegetables-like flavor notes. Despite the freeze-dried sample was brittle and crispy, firm but not too hard, airy, and easy-to-chew, the texture quality score was below 3.5 (in the range of “good” quality) compared with the scCO_2_-dried samples not pre-cooked (Flat and LT) whose texture scores were within the range of “very good” quality. These findings indicate the different behavior of red beetroot in comparison with red bell pepper and apple when exposed to freeze-drying. In both latter cases, freeze-drying resulted in the highest sensory quality scores of the dried commodities among the applied drying techniques [23,24]. As reported by the same authors, air-drying was not indicated as a good solution for the production of this kind of dried ready-to-eat fruit/vegetable snacks as it delivers products with very pronounced hardness which negatively influences chewiness, presence of off-flavors, and distinct shape deformation, which all together result in a reduction of both consumer acceptance and objective sensory quality score [22,23,24].

## 4. Conclusions

According to the sensory evaluation conducted, direct scCO_2_-drying, without a pre-cooking step and application of high temperatures, showed promising potential to be used as an alternative drying technology in the production of dried beetroot snacks, but an economic justification is required for the industrial application at a large scale. The trials with beetroot revealed that scCO_2_-drying can bring the dried product with significantly higher levels of sensory quality and consumer acceptance than can be obtained by freeze-drying which is considered today as the best drying method for many fruits and vegetables. Among the tested beetroot samples, a “very good” level of overall sensory quality was found only in those two samples dried using scCO_2_ without applying the thermal treatment before drying. Mean overall sensory quality score for freeze-dried beetroot was in the range of “good” quality. Further, almost 59% of tested consumers showed a preference for the two scCO_2_-dried beetroot samples not subjected to the cooking step before drying, which were characterized by typical beetroot magenta color, low level of shape and surface deformations, pronounced brittleness and crispiness, and good rehydration during mastication. Freeze-drying had negative effects primarily on appearance attributes, but also on the flavor of dried beetroot, resulting in partial loss of the typical beetroot flavor. The main defect in the fried beetroot was related to overburnt/grime flavor notes and appearance. Nevertheless, according to the acceptance testing, the consumers from all of the obtained clusters scored the fried beetroot product as acceptable. This could be explained by the fact that the product was salted and relatively similar to commercial potato chips or extruded corn snacks—the products that are ready-to-eat and that are commonly consumed as a fast food within the population tested in this study (university students).

## Figures and Tables

**Table 1:** The list of sensory properties and descriptors used in the descriptive sensory evaluation of dried beetroot samples.

Attribute	Definition	Anchors
**APPEARANCE**		
Flesh color intensity	The intensity or color strength of the flesh.	*light-dark*
Surface deformation	Presence of furrows, cracks, or holes on the flesh surface.	*none-lots of*
Degree of shape deformation	Deformation of the shape in relation to the flat disc (distortion, bending).	*none-much*
**ODOR** (orthonasal olfaction)		
Overall odor intensity	The intensity of the overall odor of the product.	*none-intensive*
Beetroot odor	The intensity of the beetroot-like odor.	*none-intensive*
Hay-like odor	The intensity of the hay-like and dried vegetables-like odor.	*none-intensive*
Musty odor ^1^	The intensity of odor associated with a damp cellar, or stale vegetables.	*none-intensive*
Oil odor	The intensity of the fried vegetable oil odor.	*none-intensive*
Nasal pungency	The intensity of nasal pungent and sourish feelings.	*none-intensive*
**FLAVOR**		
Overall flavor intensity	The intensity of the overall flavor of the product.	*none-intensive*
Beetroot flavor	The intensity of the beetroot-like flavor.	*none-intensive*
Sweetness	The taste stimulated by sugars.	*none-intensive*
Overburnt/Grime flavor	The intensity of the overburnt, grime-like flavor.	*none-intensive*
Bitterness	The taste stimulated by chemical substances such as caffeine or quinine.	*none-intensive*
**TEXTURE**		
Flesh surface roughness	Degree to which the surface is uneven, related to the amount of irregularity, bumps, grains, or protrusions present on the surface.	*smooth-rough*
Hardness	The force required to bite through the dried beetroot disc.	*soft-hard*
Cohesiveness	Amount of product that deforms rather than breaks during chewing.	*breaks-deforms*
Denseness ^1^	Cross-section compactness.	*airy-dense/compact*
Crispiness	The force and noise with which the beetroot slice breaks during chewing.	*not crispy-very crispy*
Adhesiveness	Degree to which mass sticks to the teeth.	*not sticky-very sticky*

^1^ These attributes were excluded from further multivariate statistical analysis because 3-way ANOVA showed they did not significantly discriminate (*p* < 0.05) among the tested samples.

**Table 2:** Consumer hedonic acceptance ^1^ for dried beetroot samples.

Beetroot Samples	Consumers ^2^ (N = 102)		
	Cluster 1 (41.2%)	Cluster 2 (33.3%)	Cluster 3 (25.5%)
**Overall Liking**			
CO_2_-dried Flat	5.9 ± 2.8 ^b,B,C^	4.3 ± 2.1 ^a,A^	7.5 ± 1.3 ^c,C^
CO_2_-dried LT	5.2 ± 2.5 ^a,A,B,C^	7.5 ± 1.5 ^b,C^	4.0 ± 2.0 ^a,A^
CO_2_-dried HT	4.0 ± 2.2 ^a,A^	5.0 ± 2.2 ^a,b,A,B^	5.7 ± 1.8 ^b,B^
Fried	6.6 ± 2.7 ^C^	6.5 ± 2.7 ^B,C^	7.3 ± 2.1 ^C^
Freeze-dried	4.9 ± 2.6 ^A,B^	5.2 ± 2.6 ^A,B^	4.6 ± 2.0 ^A,B^
**Appearance Liking**			
CO_2_-dried Flat	7.3 ± 2.1 ^a,b,C^	6.7 ± 1.9 ^a,B,C^	8.2 ± 1.1 ^b,B^
CO_2_-dried LT	6.6 ± 2.4 ^a,B,C^	7.4 ± 1.5 ^b,C^	5.5 ± 2.3 ^a,b,A^
CO_2_-dried HT	5.8 ± 2.8 ^A,B^	5.9 ± 2.3 ^A,B^	6.5 ± 2.1 ^A^
Fried	5.9 ± 2.7 ^A,B,C^	5.7 ± 2.6 ^A,B^	5.9 ± 2.5 ^A^
Freeze-dried	5.0 ± 2.5 ^A^	4.7 ± 2.6 ^A^	4.9 ± 2.4 ^A^
**Flavor Liking**			
CO_2_-dried Flat	5.5 ± 2.7 ^a,b,B^	4.8 ± 2.2 ^a,A^	6.6 ± 2.1 ^b,B^
CO_2_-dried LT	5.1 ± 2.4 ^a,B^	7.1 ± 1.8 ^b,C^	4.5 ± 2.4 ^a,A^
CO_2_-dried HT	3.1 ± 2.4 ^a,A^	4.8 ± 2.3 ^b,A^	5.0 ± 2.2 ^b,A,B^
Fried	6.2 ± 3.1 ^B^	6.5 ± 2.9 ^B,C^	6.7 ± 2.7 ^B^
Freeze-dried	5.1 ± 2.8 ^B^	5.1 ± 2.8 ^A,B^	4.4 ± 2.9 ^A^

^1^ Arithmetic mean ± standard deviation. The same lowercase letter within a row and the same uppercase letter within a column indicate values that are not statistically different (α = 0.05). ^2^ K-means cluster analysis of the consumer PCA scores.

**Table 3:** Sensory quality scores ^1^ for dried beetroot samples.

Beetroot Samples	Overall Quality	Appearance	Odor	Texture	Flavor
CO_2_-dried Flat	3.7 ± 0.6 ^b^	4.3 ± 0.6 ^c^	2.6 ± 1.2 ^a,b^	3.9 ± 0.7 ^a,b^	4.0 ± 0.7 ^b^
CO_2_-dried LT (Curvy)	3.7 ± 0.6 ^b^	3.9 ± 0.7 ^c^	2.5 ± 1.3 ^a^	4.2 ± 0.8 ^b^	3.9 ± 0.8 ^b^
CO_2_-dried HT (Curvy)	2.9 ± 0.6 ^a^	3.8 ± 0.8 ^c^	2.3 ± 0.8 ^a^	3.4 ± 0.8 ^a^	2.4 ± 1.0 ^a^
Fried	3.0 ± 0.9 ^a^	2.8 ± 0.7 ^b^	2.3 ± 1.2 ^a^	4.1 ± 0.8 ^b^	2.6 ± 1.4 ^a^
Freeze-dried	3.0 ± 1.0 ^a^	1.8 ± 1.1 ^a^	3.2 ± 1.1 ^b^	3.4 ± 0.9 ^a^	2.9 ± 1.4 ^a^

^1^ Arithmetic mean ± standard deviation (10 assessors × 2 replications). The same letter within a column indicates values that are not statistically different (α = 0.05).
